# The role of the autonomic nervous system in Tourette Syndrome

**DOI:** 10.3389/fnins.2015.00117

**Published:** 2015-05-27

**Authors:** Jack Hawksley, Andrea E. Cavanna, Yoko Nagai

**Affiliations:** ^1^North Essex Partnership University NHS Foundation TrustColchester, UK; ^2^Department of Neuropsychiatry, Birmingham and Solihull Mental Health NHS Trust and School of Clinical and Experimental Medicine, University of BirminghamBirmingham, UK; ^3^Department of Clinical Medicine, Clinical Imaging Sciences Center, Brighton and Sussex Medical School, University of SussexBrighton, UK; ^4^Department of Clinical and Experimental Epilepsy, Institute of Neurology, University College LondonLondon, UK

**Keywords:** Tourette Syndrome, autonomic nervous system, sympathetic activity, ticks, autonomic modulation

## Abstract

Tourette Syndrome (TS) is a neurodevelopmental disorder, consisting of multiple involuntary movements (motor tics) and one or more vocal (phonic) tics. It affects up to one percent of children worldwide, of whom about one third continue to experience symptoms into adulthood. The central neural mechanisms of tic generation are not clearly understood, however recent neuroimaging investigations suggest impaired cortico-striato-thalamo-cortical activity during motor control. In the current manuscript, we will tackle the relatively under-investigated role of the peripheral autonomic nervous system, and its central influences, on tic activity. There is emerging evidence that both sympathetic and parasympathetic nervous activity influences tic expression. Pharmacological treatments which act on sympathetic tone are often helpful: for example, Clonidine (an alpha-2 adrenoreceptor agonist) is often used as first choice medication for treating TS in children due to its good tolerability profile and potential usefulness for co-morbid attention-deficit and hyperactivity disorder. Clonidine suppresses sympathetic activity, reducing the triggering of motor tics. A general elevation of sympathetic tone is reported in patients with TS compared to healthy people, however this observation may reflect transient responses coupled to tic activity. Thus, the presence of autonomic impairments in patients with TS remains unclear. Effect of autonomic afferent input to cortico-striato-thalamo-cortical circuit will be discussed schematically. We additionally review how TS is affected by modulation of central autonomic control through biofeedback and Vagus Nerve Stimulation (VNS). Biofeedback training can enable a patient to gain voluntary control over covert physiological responses by making these responses explicit. Electrodermal biofeedback training to elicit a reduction in sympathetic tone has a demonstrated association with reduced tic frequency. VNS, achieved through an implanted device that gives pulsatile electrical stimulation to the vagus nerve, directly modulates afferent interoceptive signals. The potential efficacy of biofeedback/VNS in TS and the implications for understanding the underlying neural mechanisms of tics will be discussed.

## Introduction

Tourette Syndrome (TS) is a neurodevelopmental disorder in which both neurological and psychological factors underlie the expression of tics. The syndrome is characterized by multiple involuntary movements (motor tics) and vocal (phonic) tics that last more than 1 year. Recent research has shown that TS is more common than historically estimated, affecting between 0.3 and 0.4% of children worldwide, with some reports indicating a prevalence of up to one percent of the general population (Robertson, 2008a,b). TS has been identified and labeled in all countries and across racial and ethnic groups (Robertson et al., 2009). TS is more common in children, four times more common in males, and more prevalent in special educational needs settings (Comings and Comings, 1990). Perinatal insults (e.g., birth injuries) and infections may increase the incidence of TS. The presentation of tics can be diverse and symptoms fluctuate in severity with occasional remissions. This often causes problems for assessment and intervention. Starting in childhood, symptoms usually reach peak level by the age of 11 or 12 years old. Generally symptomology improves with increase in age (Robertson, 2000; Hassan and Cavanna, 2012). Most tics are either semi-voluntary or involuntary. Patients commonly experience uncomfortable premonitory sensations or urges which are only relieved after the tic action has been performed (Rajagopal et al., 2013; Crossley et al., 2014). Jankovic (1997) provides a summary of tic presentations as: temporarily suppressible; suggestible; increased by stress; increased with relaxation after stress; decreased by distraction and concentration; persisting during sleep. A high proportion of patients (as high as 80%) report premonitory sensations prior to tic expression (Cohen and Leckman, 1992). A significant proportion of patients with TS also suffer from comorbid psychological and psychiatric conditions such as Attention Deficit Hyperactive Disorder (ADHD), Obsessive Compulsive Disorder (OCD), depression and impulse control disorders (Robertson, 2000; Cavanna and Rickards, 2013). Up to 80% of patients with TS may also suffer from OCD (Eddy and Cavanna, 2014), highlighting what is thought to be a shared neural mechanism (dysfunction of caudate nucleus and anterior cingulate) which will be discussed in the later section.

One of the arguments for implicating the autonomic nervous system (ANS) in the etiology and maintenance of TS is that, tic activity is linked to a patient's psychological and emotional state (Steinberg et al., 2013a,b), and that sympatholitic drugs such as Clonidine are often used as the first line medication to treat patients with TS (Cavanna et al., 2012). Peripheral modulation of sympathetic nervous system also influences tic expression (Nagai et al., 2009). In the current manuscript, we explore and discuss the relatively under-investigated area of the role of autonomic function on motor activity, especially focusing on interaction between autonomic activity and tics in patients with TS.

## Autonomic activity and Tic expression

A relationship between tic frequency and bodily states of arousal has been observed (Nagai et al., 2009). The tic activity was observed during experimental induction of relaxation or arousal using electrodermal biofeedback (Nagai et al., 2009). Electrodermal activity (EDA) reflects the sympathetic nervous activity of sweat glands. Patients produced significantly fewer tics during relaxation biofeedback (decrease in skin conductivity) compared to the arousal biofeedback (increase in skin conductivity). TS patients commonly report symptoms, which are consistent with sympathetic over-activity: increased heart rate, body temperature, nervousness, and agitation (Shapiro et al., 1988). Relaxation training is incorporated into some therapeutic training (Azrin and Peterson, 1988) however the use of relaxation therapy on its own has been shown not to be effective in reducing the frequency of tics (Bergin et al., 1988). Relaxation therapy is often composed of reducing muscular tension and breathing manipulation, however they usually lack the monitoring of physiological measures. Thus, main mechanisms of therapeutic effect are often unclear. Autonomic modulators are used as a first line medication to reduce symptom of TS. Clonidine (an alpha-2 adrenergic agonist) which inhibits release of noradrenalin and suppresses sympathetic activity is often prescribed for children with TS due to its tolerability of side effect and also due to caution of long term side effect with dopaminergic agents (Goetz, 1992). By stimulating alpha-2 receptors in the vasomotor centers within the brain stem, the release of norepinephrine is inhibited with an overall effect in decreasing sympathetic tone (British National Formulary, 1998). Clonidine is also effective in reducing symptoms of ADHD and OCD, both comorbidities of TS, as well as reducing irritability, aggressiveness, frustration tolerance, oppositional behaviors and improving interpersonal functioning (Cohen et al., 1980). Clinical trials of clonidine report mixed results, with some studies supporting efficacy (e.g., Leckman et al., 1982, 1991; Singer et al., 1985; Hedderick et al., 2009) while others have argued against the effectiveness (e.g., Singer et al., 1985; Goetz et al., 1987).

Tetrabenazine (Vesicular monoamine transporter: VMAT inhibitor) has been used as an effective treatment for refractory TS (Jankovic and Beach, 1997). VMAT2 plays an important role in transporting norepinephrine and other monoamines in synaptic vesicles both in the periphery and central nervous system (Ben-Dor et al., 2007; Eiden and Weihe, 2011; Schafer et al., 2013). A significant reduction of platelet VMAT2 is reported in patients with TS indicating, if occurred in the brain, adaptive mechanisms to dopaminergic over reactivity (Ben-Dor et al., 2007). The reduction of VMAT2 also indicates impaired circulation of autonomic agent (norepinephrine).

In an attempt to implicate the parasympathetic nervous system with TS, Tanner et al. (1982) showed a reduction of motor tics and an increase of vocal tics after scopolamine injection. The scopolamine effect was reversed by an injection of physostigmine. These data suggest that the cholinergic/central parasympathetic system might be implicated in the pathophysiology of TS and that cholinergic manipulation may benefit some patients (Tanner et al., 1982). In addition, the apparent dissociation between motor and vocal tics raises the possibility that cholinergic neurotransmission might play a role in the differential expression of these two tic types. Interestingly, the results of clinical studies conducted in more recent years using hierarchical cluster analysis and principal component factor analysis techniques suggested a separation of tic symptoms, with complex vocal tics selectively segregating with socially inappropriate behaviors (Robertson et al., 2008), coprophenomena (Cavanna et al., 2011), and impulsivity (McGuire et al., 2013). Taken together, these findings prompt further research aimed at investigating whether the role of the autonomic system in the differential expression of motor and vocal tics implies the presence of multiple underlying mechanisms. Table 1 describes comparison of studies investigating autonomic functions in patients with Tourette Syndrome.

**Table 1:** **Comparison of studies investigating autonomic functions in patients with Tourette Syndrome**.

**Study**	**Autonomic and other measurements**	**Patients**	**Results**
Lake et al., 1977	Noradrenaline concentration in standing and seated position	TS patients (*n* = 33) Healthy control (*n* = 22)	No significant difference between the changes of concentration
Bock and Goldberger, 1985	Basal skin resistance levels and phasic skin resistance responses to the following tests: (1) Continuous Performance Test, (2) Habituation-sound, (3) Habituation-light, (4) Color-word test	TS patients (*n* = 20) Patients with other chronic illness (e.g., diabetes, hemophilia) (*n* = 20)	Patient group showed less change in arousal (skin resistance level) than controls during the sound and light habituation tests. No other group differences
Schelkunov et al., 1986	Heart rate and blood pressure	TS patients (*n* = 24) Patients with other conditions (*n* = 48): temporal lobe epilepsy (*n* = 20), schizophrenia (*n* = 15), residual organicity (*n* = 5), myoclonic epilepsy (*n* = 7)	The mean cardiointerval (CI) was significantly higher in patients with TS compared with patients with other conditions
Goetz et al., 1987	Pulse rate and blood pressure measurements during three tasks: (1) different body positions (supine, standing, and prolonged standing), (2) a cold presser response test (a cardiovascular test which involves immersing the hand into an ice water container), and (3) the Valsalva maneuver (exhalation against a closed airway)	TS patients (*n* = 23) Healthy control (*n* = 23)	No significant difference between patients and controls in any autonomic parameters
van Dijk et al., 1992	Heart rate measurement during (1) rest, (2) deep breathing, (3) standing up, (4) Valsalva maneuver test, (5) blood pressure test	TS patients (*n* = 18) Healthy control (*n* = 23)	Higher heart rate in patients with TS in Valsalva maneuver test. No significant difference in other autonomic parameters
Chappell et al., 1994	(1) Plasma adrenocorticotropin (ACTH), (2)cortisol, (3) urinary catecholamines, (4) anxiety level during lumbar puncture (LP) stress	TS patients (medication free) *n* = 13 Healthy control (*n* = 10)	TS patients secreted significantly more ACTH than controls in response to the stress of the lumber puncture. TS patients excreted more norepinephrine prior to lumber puncture. Urinary norepinephrine excretion of TS patients was significantly correlated with tic severity
Tulen et al., 1998	(1) Plasma catecholamines, (2) Heart rate, (3) Blood pressure during the Color Word test, preceded by a baseline measure	TS Patients (*n* = 9) Health control (*n* = 9)	Enhanced cardiovascular activity (both mean heart rate and mean blood pressure) in TS patients during sitting/rest than controls, but no differences plasma catecholamines. No significant differences in (1, 2, 3) between groups during the Color Word test
Wood et al., 2003	Heart rate and respiratory rate measured while watching a movie to elicit certain emotional states	TS Patients (*n* = 4)	Tics induced differentially by certain emotional states. For example anger and happiness produced lowest tic severity in comparison to sadness and fear which produced intermediate severity
Nagai et al., 2009	Tics were monitored during relaxation (decrease in sympathetic tone) and arousal (increase in sympathetic tone) biofeedback	TS Patients (*n* = 15)	Tics were significantly lower during relaxation biofeedback compared to arousal biofeedback. Tic frequency was positively correlated arousal level during arousal biofeedback

## Autonomic abnormalities in tourette syndrome

The extent to which the ANS is dysfunctional in TS remains uncertain, with research studies producing conflicting and inconclusive data. The inconsistency to date is influenced by several factors: differences in experimental protocols; the absence of healthy control groups in some studies; and the use of medication in patient groups. The inconsistency could also be related to the complex clinical profile of TS—with multiple co morbidities, associated behaviors, and psychopathologies which makes it difficult to isolate ANS abnormality as a distinct, let alone causal or maintaining, factor in TS. The next section will consider some of the empirical literature relating to attempts to investigate potential autonomic abnormalities in TS patients.

Lake et al. (1977) investigated changes in plasma noradrenaline concentrations in different positions (standing and seated) between TS patients and controls (Lake et al., 1977). No difference found between groups, indicating intact central mechanisms for orthostatic regulation of sympathetic activity. In a study by Bock and Goldberger (1985), basal skin resistance levels and phasic skin resistance responses were measured across TS patients and a control group during four psychological tests (the continuous performance test, habituation-sound, habituation light, and the color-word Stroop task). The patient group showed less change in electrodermal arousal than controls during the sound and light habituation tests. The finding that patients showed less habituation might reflect more persistent engagement of the reticular activating systems in these patients (Bock and Goldberger, 1985). Contrary to most research, Schelkunov et al. (1986) reported slower and more variable heart rate and reduced blood pressure, suggesting increased parasympathetic tone.

One investigation of the ANS using non-invasive measurements of pulse rate and blood pressure during three tasks: different body positions (supine, standing, and prolonged standing), cold pressor response test (a cardiovascular test which involves immersing the hand into an ice water container), and the Valsalva maneuver (exhalation against a closed airway) showed no difference in TS patients vs. controls (Goetz et al., 1987).

van Dijk et al. (1992) investigated ANS function in 18 TS patients and 23 matched controls using four heart rate tests: variation at rest, during deep breathing, following standing up, and during a Valsalva maneuver and two blood pressure tests. The only significant difference between patients and controls was in the Valsalva test. The patient group showed larger maximum, but not minimum heart rates compared to the controls (*P* = 0.03). This was due to the initial heart rate in this group of TS patients being higher than the control group. This suggests that there is a possible implication of sympathetic activity on tics, however this was not be supported by the other five autonomic tests used in the study.

In their study, Chappell et al. (1994) investigated the effects of stress caused by the anticipation of a lumbar puncture operation on plasma adrenocorticotropin (ACTH) and cortisol, urinary catecholamines, and ratings of anxiety. They concluded that TS patients may be subject to heightened reactivity of the HPA axis and associated noradrenergic sympathetic systems. They found that the TS patients secreted significantly more ACTH in response to the lumbar puncture and excreted significantly more norepinephrine than controls when anticipating the operation. Tics increased with stress, fatigue, emotional trauma, and anxiety (Chappell et al., 1994). This is in accordance with data from a small scale pilot study in which emotional arousal was induced in four children with TS by watching a movie known to engender specific emotional states (Wood et al., 2003). In this study, tic severity did not correlate with heart or respiratory rate. Instead the authors posited that tics, rather than being autonomically mediated, were induced differentially by certain emotional states. For example anger and happiness produced lower tic severity in comparison to sadness and fear, which produced intermediate severity. The stress response in TS is well summarized in the recent paper of Martino et al. (2013). The review considers the involvement of neuroendocrine mechanisms (involving both sex and stress steroid hormones) to the understanding of TS. The drugs counteracting the effect of testosterone and possibly the neuropeptide oxytocin may alter tics (Martino et al., 2013). It also describes the role of neuroendocrine system (Hypothalamus, Pituitary gland, and Adrenal cortex: HPA axis) to stress responses in parallel with autonomic reactions.

In a study looking at cardiovascular and catecholaminergic activity during mental load, Tulen et al. (1998) pointed to the functional complexity of the ANS in TS. Their findings again indicated enhanced cardiovascular activity in the patients, with higher heart rate and blood pressure during baseline compared to healthy controls. They found that a significant difference to controls was not maintained during the mental load task. There was no significant difference in plasma catecholamines, based on blood samples taken before and during the task. They concluded that there was some evidence of increased sympathetic activity in the TS group and no evidence for alterations in parasympathetic activity in the patient group (Tulen et al., 1998).

Overall these studies provide variable support for the notion that both the sympathetic and parasympathetic nervous systems can influence tic expression and frequency. However, data are not conclusive, possibly reflecting limitations of experimental design and the functional complexity of TS, which makes it difficult to differentiate patients' autonomic functioning from healthy controls.

## Central Tic regulation and autonomic nervous system

The precise neural mechanisms of tic generation are still to be clarified. However, it is generally agreed that tics are caused by a failure of inhibitory action within a cortico-striato-thalamo-cortical circuit that suppresses premonitory urges for movement (Mink, 2001; Albin and Mink, 2006). This circuit in frontal cortex, basal ganglia, and thalamus plays an important role in coordination and fine motor control. Groups of subcortical neurons within the basal ganglia translate top-down motor control into inhibitory action of the striatum (caudate and putamen) and globus pallidus on the thalamus, which in turn sends information back to the cortex. Recent neuroimaging investigations indeed revealed overall increases in activation within motor pathways in patients with TS compared to healthy controls mimicking tic behavior, although activity within anterior cingulate, caudate, and parietal operculum was significantly reduced, indicating a lack of inhibitory motor control (Wang et al., 2011).

**Figure 1:**
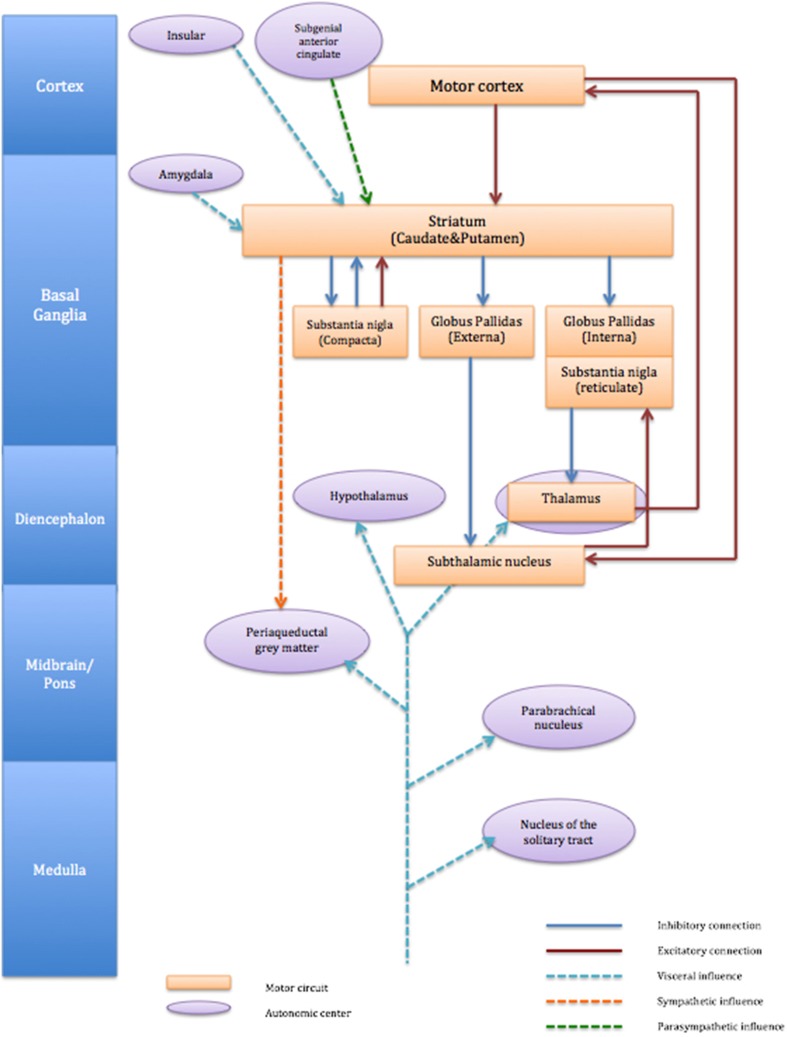
**Schematic connection of cortico-striato-thalamo-cortical circuit and associated autonomic centers**. The figure describes cortico-striato-thalamo-cortical circuit (orange box) with excitatory connection (red line) and inhibitory connection (blue line). Autonomic centers (purple circle) influences striatum are visceral input from amygdala and insula (light blue dot line) and parasympathetic influence from subgenial anterior cingulate (green dot line). Striatum also exert influence to sympathetic (orange dot line) periaqueductal gray matter.

Autonomic afferents fibers carry visceral information from almost all the organs and ascend through the vagus nerve and spinal cord (Janig, 2006). Such peripheral afferent information is processed centrally and can create sensations that motivate behavior according to necessity, e.g., thirst, hunger, pain. The direct projections of afferent visceral nerves terminate either at nucleus of the solitary tract (nucleus tractus solitarii, NTS), parabrachial nucleus or thalamus (ventromedial posterior thalamus) and information is further carried at basal ganglia and cortical levels (Critchley and Harrison, 2013). The influence of autonomic activity on motor function is not clearly understood. However, it is worth noting that within cortico-striato-thalamo cortical circuits, the striatum receives afferent information directly from both right and left amygdala and viscerosensory insular cortex. It is also derived by cortical centers implicated in parasympathetic regulation for example the subgenual anterior cingulate (Critchley and Harrison, 2013). However, autonomic outputs from the striatum appear dominated by sympathetic drive linked to motor function (see Figure 1). We discussed in the previous section that autonomic dysfunction in patients with TS is difficult to conclude unlike some neurological condition (sudden unexpected death in epilepsy: SUDEP) with which autonomic dysfunction can be fatal. It is likely that emotional disturbance influences striatal activity through visceral input of amygdala and insular (Figure 1). Impairment of inhibitory mechanisms within cortico-striato-thalamo cortical circuit may thus underlie uncontrollable motor tics in TS.

## Autonomic modulation as a treatment of TS

Modulation of the autonomic nervous system can be achieved through vagus nerve stimulation (VNS) and autonomic biofeedback. VNS uses an implanted device for pulsatile electrical stimulation of the vagus nerve, directly modulates afferent interoceptive signals. Two small scale studies have applied VNS to TS (Diamond et al., 2006; Sperling et al., 2008). In the single case report by Diamond et al. (2006) an implanted vagal nerve stimulator led to improvements on the total tic score and the motor and phonic tic frequency. One study also reported improvement in tic severity and clinical ratings following VNS, however the effectiveness of this treatment needs to be confirmed by systematic clinical trials with an appropriate sample size (Sperling et al., 2008). Nagai et al. (2009) investigated the efficacy of electrodermal biofeedback on tic activity in patients with TS. Biofeedback is a non-invasive psychophysiological intervention, which can be used to directly modulate physiological responses. It enables patients to perceive their bodily signals through visual and auditory feedback and eventually to learn to control their bodily state of arousal. The investigation using electrodermal biofeedback training, targeting sympathetic arousal level, have found that a reduction in sympathetic tone was associated with a reduction in tic frequency (Nagai et al., 2009). However, contrary to the findings in a basic study where patients showed significantly less tic activity during 5 min of relaxation biofeedback, interpretation of the clinical trial was not straightforward that patients found it difficult to maintain longer period of relaxation biofeedback as a treatment due to concomitantly occurring tics (Nagai et al., 2014).

## Conclusion

There is a paucity of research regarding autonomic influences on tics. The central (efferent) influence of tic activity on peripheral autonomic activity remains unclear due to inconclusive results of the different studies, however it is likely that afferent visceral input influences cortico-striato-thalamo-cortical circuit through enhanced dysfunction of inhibitory mechanisms of striatum. Understanding the relationship between peripheral autonomic activity and central motor activity is important for elucidating how emotional disturbance affect tic activity (visceral afferent input as a trigger of tics) and for investigating how peripheral autonomic modulation may contribute to the treatment of TS.

### Conflict of interest statement

The authors declare that the research was conducted in the absence of any commercial or financial relationships that could be construed as a potential conflict of interest.
